# Processes in auroral oval and outer electron radiation belt

**DOI:** 10.1186/s40623-018-0898-1

**Published:** 2018-08-08

**Authors:** Elizaveta E. Antonova, Marina V. Stepanova, Pablo S. Moya, Victor A. Pinto, Vadim V. Vovchenko, Ilya L. Ovchinnikov, Nikita V. Sotnikov

**Affiliations:** 1 Skobeltsyn Institute of Nuclear Physics and Space Research Institute RAS, Lomonosov Moscow State University, Moscow, Russia; 2 Departamento de Fisica, Universidad de Santiago de Chile (USACH), Santiago, Chile; 3 Departamento de Fisica, Facultad de Ciencias, Universidad de Chile, Santiago, Chile; 4 Department of Atmospheric and Oceanic Sciences, University of California, Los Angeles, USA; 5 Space Research Institute RAS, Moscow, Russia; 6 Skobeltsyn Institute of Nuclear Physics, Lomonosov Moscow State University, Moscow, Russia

**Keywords:** Magnetospheric storm and substorm, Auroral oval, Acceleration of electrons of the outer electron radiation belt

## Abstract

We have analyzed the role of auroral processes in the formation of the outer radiation belt, considering that the main part of the auroral oval maps to the outer part of the ring current, instead of the plasma sheet as is commonly postulated. In this approach, the outer ring current is the region where transverse magnetospheric currents close inside the magnetosphere. Specifically, we analyzed the role of magnetospheric substorms in the appearance of relativistic electrons in the outer radiation belt. We present experimental evidence that the presence of substorms during a geomagnetic storm recovery phase is, in fact, very important for the appearance of a new radiation belt during this phase. We discuss the possible role of adiabatic acceleration of relativistic electrons during storm recovery phase and show that this mechanism may accelerate the relativistic electrons by more than one order of magnitude. 
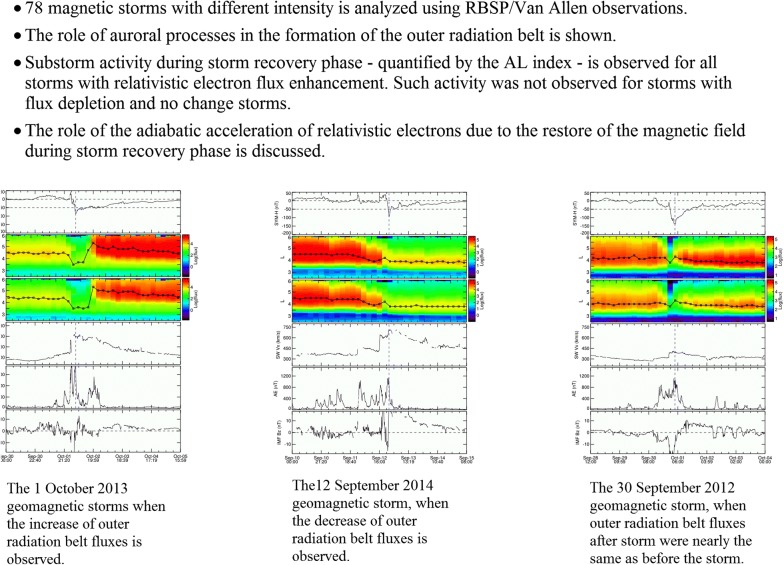

## Introduction

It is well known that during large geomagnetic storms, bright discrete auroral forms are observed at low latitudes. This phenomenon is connected to the expansion of the storm time auroral oval to lower latitudes. Simultaneously, we observe the development of the ring current. For a long time, it was suggested that the auroral oval maps into the plasma sheet. However, starting from early measurements (Frank 1971), it is known that around midnight the plasma sheet and ring current regions partially overlap. It was also known that plasma sheet-like plasma surrounds the Earth, forming the plasma ring (Paschmann et al. 2002; Antonova et al. 2013, 2014). The existence of this ring leads to the idea of mapping the auroral oval to the plasma ring instead the plasma sheet. However, most of the magnetic field models are based on a predefined geometry of currents and are overstretched (see the discussion in Antonova et al. 2017). Therefore, it was necessary to use “topological mapping” to prove this hypothesis. The “topological mapping” is based on the conservation of definite plasma parameters along a magnetic field line. Antonova et al. (2015) used the plasma pressure for this purpose, which is conserved for plasmas with isotropic pressure in magnetostatic equilibrium (when sound and Alfvén velocity are much larger than the plasma velocity). They showed that most of the auroral oval is mapped to the surrounding the Earth plasma ring. Studies of transverse currents in this ring showed that they surround the Earth and close inside the magnetosphere (Antonova et al. 2017). Such findings contradict the generally accepted point of view but could be useful to solve many long-standing magnetospheric problems.

One of such long-standing problems in magnetospheric physics is the acceleration of relativistic electrons in the outer radiation belt (ORB). Acceleration of electrons during magnetic storms is well established (see Reeves 1998; Tverskaya 2011 and multiple other works). However, strong substorm activity without storms can also lead to relativistic electron enhancements (Kim et al. 2015; Hajra et al. 2015; Pinto et al. 2018). These results clearly show that auroral processes cannot be ignored in the analysis of ORB electron acceleration and that relativistic electrons can be accelerated rather quickly on the timescales of auroral substorms. The relation between the auroral processes and the ORB dynamics is easier to understand if we consider that the auroral oval maps to the outer part of the ring current. The details of this relation will be discussed in the next section.

It is known that storm time acceleration of outer belt electrons starts with the formation of a “seed” population during storm time substorms (see, for example Baker et al. 2005). It is generally suggested that this seed population is later accelerated due to wave–particle interactions, with different wave modes producing powerful fluxes of relativistic electrons. Such suggestion is based on the simultaneous observations of relativistic electrons and VLF and ULF waves. The most developed model considers whistler-mode chorus waves as a main source of acceleration. However, such processes strongly depend on the amplitudes of the observed waves and require comparatively long time, which is much larger than the timescale of substorms. In particular, Horne et al. (2005) estimated the time needed to increase the flux of 1 MeV electrons by an order of magnitude by whistler-mode chorus waves and concluded that such time is approximately 1 day. Similarly, Thorne et al. (2013) and Li et al. (2014) obtained that the time for such kind of acceleration is about 12 h. The theory is based on the suggestion that the process of acceleration may be described as the diffusion using the two-dimensional Fokker–Planck equation. The developed model can reproduce observed timing, magnitude, energy and pitch angle distribution of relativistic electron phase space density (PSD) obtained using Van Allen Probes observations and Tsyganenko and Sitnov (2005) model of storm time magnetic field distribution. However, the developed model does not take into account possible contribution of nonlinear processes (see, for example Omura and Summers 2006; Demekhov et al. 2006; Shklyar 2017) and the coincidence of the region of ORB acceleration with the position of the auroral oval (Antonova and Stepanova 2015).

It is also well known that during storms and large substorms, the whole auroral oval is filled by electrostatic and electromagnetic fluctuations with large amplitudes at different frequency ranges. Therefore, the simultaneous observations of chorus waves and relativistic electrons may mean that both phenomena develop in the same region but not necessarily have a cause–effect relationship. This is why it is interesting to analyze other possibilities. For example, Shklyar and Kliem (2006) showed that interactions of relativistic electrons with upper hybrid waves could significantly change the electron dynamics. Variations of the magnetic field inside the ring current region are also not well known yet. Kim and Chan (1997) studied the role of purely adiabatic processes (assuming the conservation of all three adiabatic invariants) for storm time relativistic flux dynamics and showed that the simple conservation of these invariants during storms can explain most drops in the relativistic electron fluxes observed at geosynchronous orbit during storms. They calculated that the decrease in the magnetic field during the main phase of the storm equivalent to Dst = − 100 nT produces a decrease in up to two orders of magnitude in the relativistic electron flux. However, the obtained result strongly depends on the magnetic field model in use. Nevertheless, it clearly indicates that the impact of magnetic field variations on the dynamics of relativistic electrons cannot be neglected. For example, it is possible to suggest that the injection of electrons with a power-law energy spectrum in the region of depressed by ring current magnetic field can lead to the appearance of large fluxes of relativistic electrons when the magnetic field restores after the storm.

Such possibility was discussed by Tverskoy (1997) and Antonova (2006). They proposed that the fluxes of ORB electrons can increase due to substorm injections of seed population electrons into the region of the magnetic field depressed by the storm time ring current. Later these electrons are adiabatically accelerated during the storm recovery phase when the magnetic field turns back to the pre-storm level. The Tverskoy’s (1997) theory was developed to explain the empirical relation between the maximum absolute value of the Dst variation during the storm (max|Dst|) and the *L*-shell location of the maximum flux of the outer radiation belt *L*_max_ in *R*_E_ after the storm (considered, as the peak intensity of relativistic electron flux with energy ~ 1 MeV). This dependence was first obtained by Tverskaya (1986) and has the form |Dst|_max_ = *c* (*L*_max_)^−4^. Here c is the coefficient of proportionality equal to 2.75 × 10^4^ nT. The use of the SYM-H index instead of the Dst gives *c* = 3 × 10^4^ nT (Tverskaya 2011). It is necessary to mention that Tverskaya’s relation has no explanation in several developed models of ORB dynamics.

The Tverskaya (1986) relation has been validated by many researches for magnetic storms with well-defined main and recovery phases (see Tverskaya 2011; Kuznetsov et al. 2002; Slivka et al. 2006; Moya et al. 2017 and references therein). Recently, Antonova and Stepanova (2015) proved this relation for the October 2012 magnetic storm, an event in which the position of the ORB maximum (maximum of the phase space density of relativistic electrons after the storm) was clearly determined by Reeves et al. (2013). Antonova and Stepanova (2015) also showed that for this storm some other important predictions are valid: a sharp peak of plasma pressure and the equatorial boundary of the westward electrojet, both located near *L*_max_. Theoretical suggestions about the role of substorm activity during storms and the action of adiabatic mechanisms of electron acceleration have not been verified yet.

In this work, we discuss the potential importance of the role of substorm activity in the electron acceleration but do not analyze it in detail. We try to verify some predictions from Tverskoy’s (1997) theory (such as the development of substorms during the storm recovery phase) as a necessary condition for the appearance of large fluxes of relativistic electrons after storms. We also try to evaluate the role of adiabatic processes in the acceleration of ORB electrons at comparatively low latitudes. The paper is organized as follows: in the data analysis section, we first examine high-energy electron fluxes and substorm activity during the storm recovery phase of 78 storms, using data of the Van Allen Probes mission. We then analyze the variations of relativistic electron fluxes for magnetic storm where the electron flux after the storm was similar to the pre-storm flux. Last sections are dedicated to discussion and conclusions.

### Data analysis

**Fig. 1:**
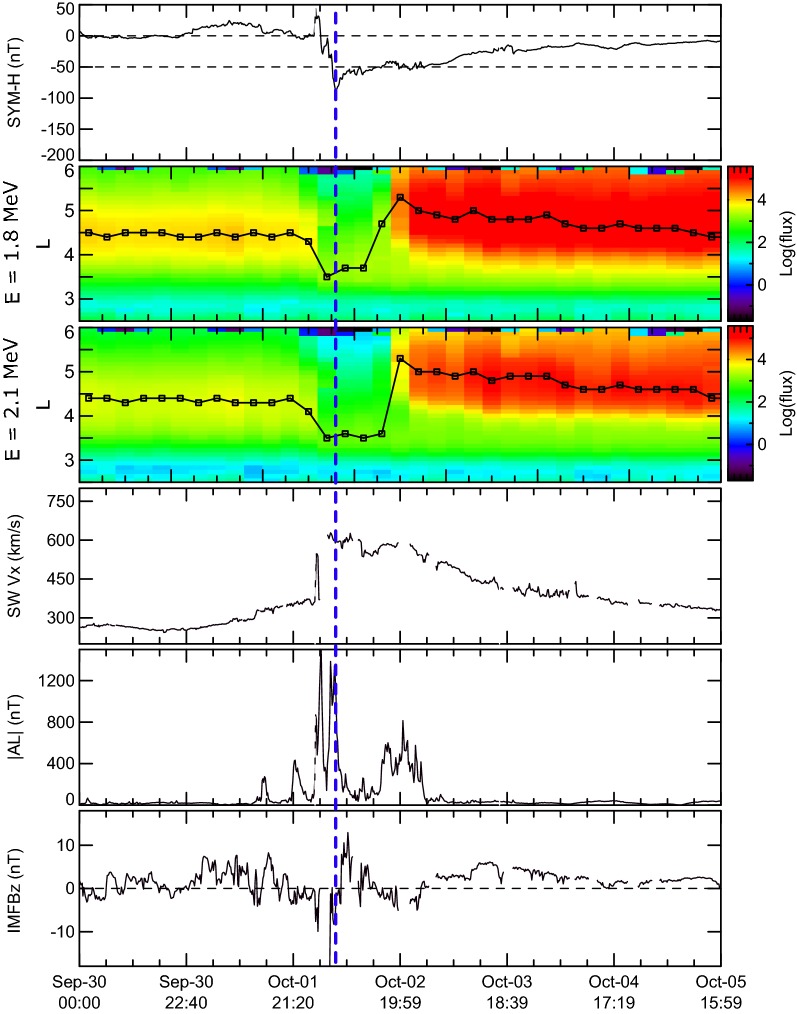
From top to bottom: **a** the SYM-H index; **b**, **c** the differential omnidirectional fluxes of 1.8 and 2.1 MeV electrons binned at 0.1 L every 4 h, respectively; **d** the solar wind speed |Vx| component; **e** the AE index; and **f** the IMF Bz component in GSM observed during 1 October 2013 geomagnetic storms when the increase in ORB fluxes is observed

**Fig. 2:**
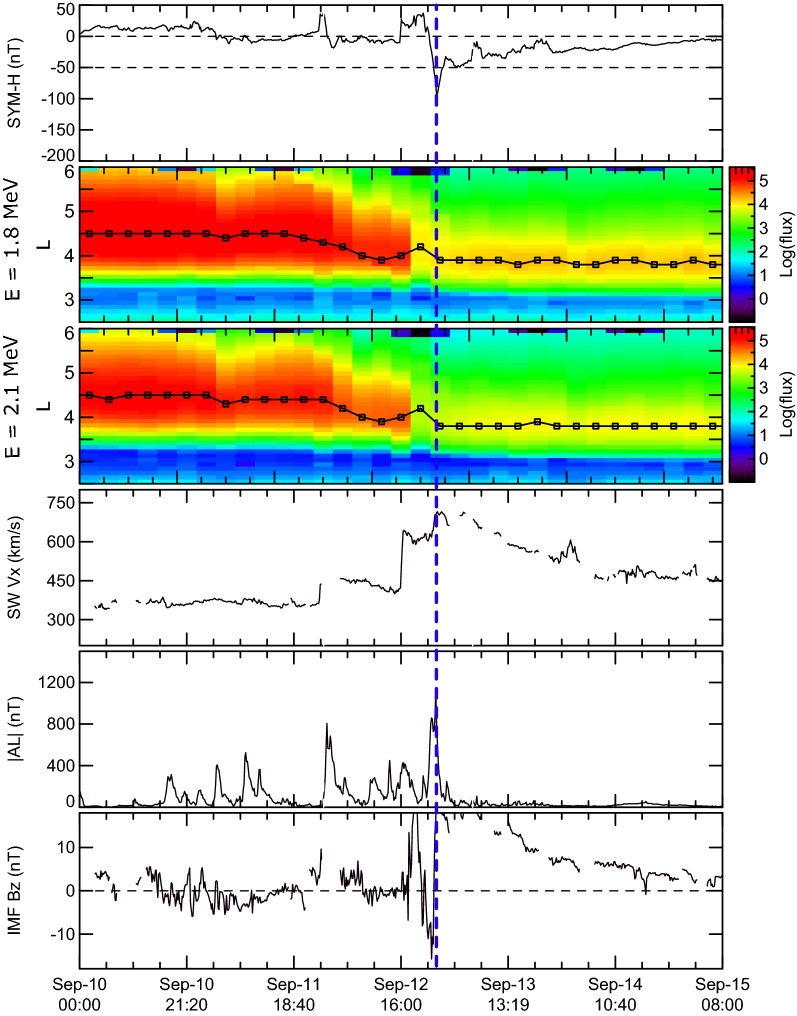
The same as in Fig. 1 for the 12 September 2014 geomagnetic storm, when the decrease in ORB fluxes is observed

**Fig. 3:**
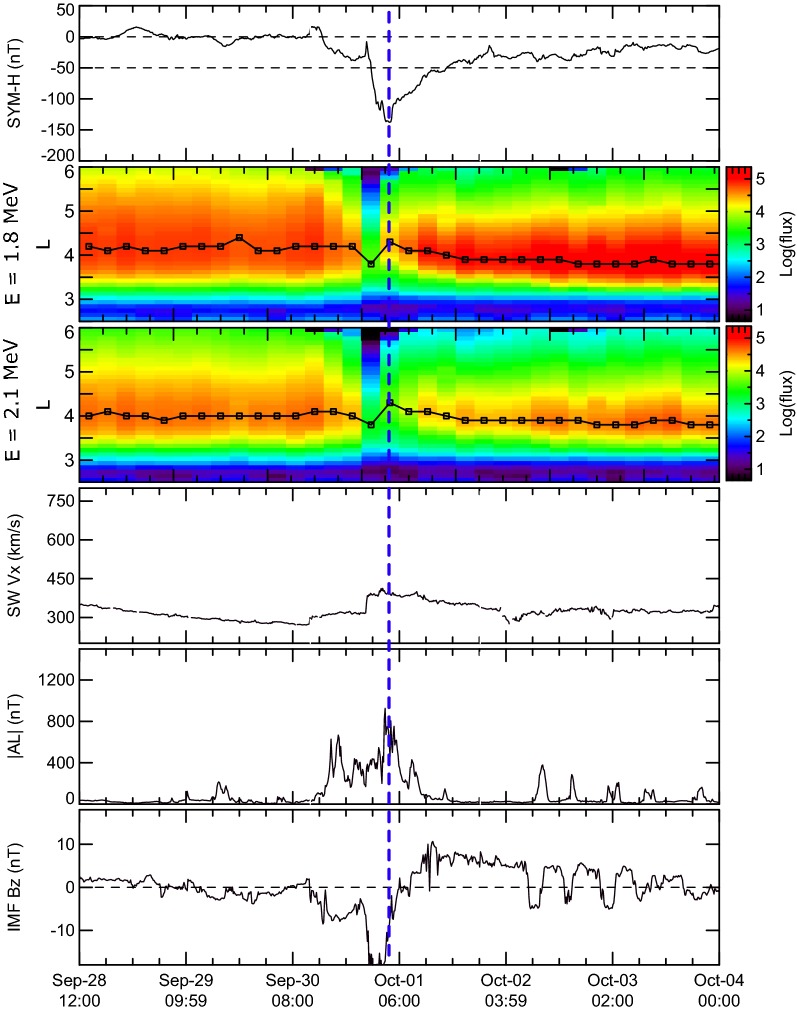
The same as in Fig. 1 30 September 2012 geomagnetic storm, when ORB fluxes after storm were nearly the same

To clarify some features of the Earth’s outer radiation belt electron acceleration, we used the list of events studied by Moya et al. (2017) that was obtained by selecting all geomagnetic storms with SYM-H minimum < − 50 nT between October 1, 2012, and June 30, 2016. Using the data of the Van Allen Probes ECT-REPT instrument (Baker et al. 2013; Spence et al. 2013) they examined the variation of MeV fluxes for 78 storms with different SYM-H following the criteria set by Reeves et al. (2003), and Turner et al. (2013), that a magnetic storm can result in an enhancement, depletion or no change in relativistic electron fluxes. Figures 1, 2 and 3 show examples of such storms and contain (from top to bottom) (a) the SYM-H index; (b) and (c) the differential omnidirectional fluxes of 1.8 and 2.1 MeV electrons binned at 0.1 *L* every 4 h, respectively; (d) the solar wind speed |Vx| component; (e) the |AL| index; and (f) the IMF Bz component in GSM. The solar wind parameters and IMF data were obtained from the OMNI dataset available at NASA’s CDAWeb repository (https://cdaweb.sci.gsfc.nasa.gov). Black line on panels (b) and (c) indicate the *L*-shell with maximum electron flux at any given time bin during each event. We are expanding the analysis in Moya et al. (2017) by focusing on the presence or absence of substorm activity during storm recovery phases. Vertical blue dashed line marks the time of SYM-H minimum.

Depletion of electron fluxes during the storm main phase is observed for the 01 October 2013 geomagnetic storm with minimum SYM-H = − 90 nT (Fig. 1) followed by flux enhancement during storm recovery phase. This storm can be considered as the first type magnetic storm. Such increase is practically coinciding with a comparatively large substorm activity as measured by AL index. It is possible to see that the flux dropout takes place during storm main phase when great substorms with |AL| index larger than 1000 nT are observed and continues during early recovery. Sharp increase in particle fluxes takes place during recovery phase substorms with maximum |AL| ~ 1000 nT, with timescale smaller than 6 h at *L* > 4. On the other hand, the position of the new formed ORB in accordance with Tverskaya relation should be located at *L* = 4.3 (for a minimum SYM-H equal to − 88 nT), which is in a rather good relation with the *L*-shell position with maximum electron flux after the storm.

In contrast, persistent depletion of relativistic electron fluxes is observed during 12 September 2014 magnetic storm, with minimal SYM-H = − 97 nT (Fig. 2). New ORB did not form after this storm instead with large magnetic substorms with |AL| up to ~ 1000 nT during the storm main phase. ORB depletion starts with the storm main phase onset, and particle fluxes do not recover to the pre-storm levels. They were nearly constant after SYM-H increased to − 50 nT (dashed line on Fig. 2b, c). The same figure shows that no substorm activity was observed during this storm recovery phase (|AL| was smaller than 200 nT).

Magnetic storm of September 30, 2012 (Fig. 3) is a storm of the third type. Particle fluxes are depleted after storm only at *L* > 4. However, at *L* ~ 4, electron fluxes were near the same after storm as before the storm. For this event, SYM-H minimum was equal to − 138 nT. This storm was analyzed by Turner et al. (2014a, b) suggesting the validity of Tsyganenko and Sitnov (2005) magnetic field model and developed model of acceleration by whistler-mode chorus waves. Low *L* losses were explained by ORB electron scattering in the loss cone by electromagnetic ion-cyclotron (EMIC) waves. The ORB losses at large *L* were explained by outward radial transport and magnetopause shadowing (Turner et al. 2014a). Stable character of particle fluxes at the center of ORB was not discussed by Turner et al. (2014a, b). However, analyzing figures 3b in Turner et al. (2014a) and figure 2 in Turner et al. (2014b) it is possible to see that the maximum of the calculated PSD practically coincides when comparing before the storm (at 30 Sep: 05:30 UT) and after the storm (at 2 Oct: 05:30 UT). In accordance with Turner et al. (2014b), “there was very little substorm activity during the storm recovery phase.” Substorm activity is observed at the beginning of the recovery phase. It is practically stopped after ~ 12:00 UT on 1 October, which is also supported by absence of injections on geostationary satellites and very little chorus activity (Turner et al. 2014b). However, Fig. 3 shows that substorm activity was very low (|AL| < 300 nT) even after ~ 06:00 UT on 1 October. The existence of a period with very low geomagnetic activity during a large portion of the storm recovery phase and the classification of this storm as no-change event (regarding the response of relativistic electron fluxes) allow us to analyze this event additionally to clarify the possible role of adiabatic effects (see below).

**Fig. 4:**
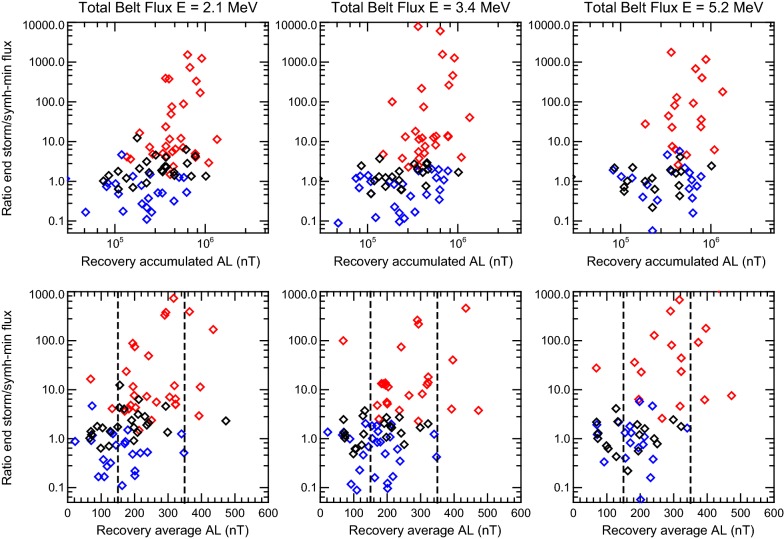
Relation between integrated |AL| index (top) and average |AL| index (bottom) during the recovery phase of each storm event, and the post-storm/minimum flux ratio integrated between *L* = 3 and *L* = 6, for *E *= 2.1 MeV (left), *E* = 3.4 MeV (center) and *E* = 5.2 MeV (right) electron fluxes. Red, blue and black squares represent enhancement, depletion and no-change events, respectively. Vertical dashed lines separate the distribution in three groups, according to their average |AL| index

We checked all 78 storms from Moya et al. (2017) database and encounter the same features. Increase in relativistic electron fluxes takes place when storm time substorms are observed during storm recovery phase. In Fig. 4, we show a comparison (between all 78 events) of the ratio between the ORB total electron flux (the integrated flux between *L* = 3 and *L* = 6) after the storm and at the moment with the minimum SYM-H index (usually the minimum flux measured during a storm) and the substorm activity during the recovery phase, measured by the |AL| index, for different energies. Top panels show the post/minimum integrated flux versus the integrated |AL| index during the recovery phase, and bottom panels show the post/minimum flux ratio versus average |AL| index during the recovery phase. In addition, following Moya et al. (2017), red, blue and black squares represent enhancement, depletion and no-change storm. Vertical dashed lines separate the distribution in three groups, according to their average |AL| index. The first group corresponds to events in which average |AL| < 150 nT, which consist (at 2.1 MeV) of 23 events, divided in 2 (9%) enhancements, 9 (39%) no-change and 12 (52%) depletion events. The second group corresponds to 48 events in which 150 nT < |AL| < 350 nT with 48% enhancements, 29% no change and 23% depletion, while group 3 (events with average |AL| > 350) contains 7 events, with 5 (71%) enhancements and 2 (19%) no-change events. Similar numbers are also found for 3.4 and 5.2 MeV channels. Such finding agrees with Tverskoy (1997) and Antonova (2006) suggestion that substorm injections during storm recovery phase can lead to appearance of powerful fluxes of relativistic electrons and that the probability of enhancement increases as average |AL| increases during the recovery phase of a storm. Interestingly, the ratio of fluxes can be relatively lower than expected for the event with the highest |AL| average, which may suggest a change in the response for extreme events. However, the number of events in group 3 is not large enough to assure such conclusion. A different statistical study will be needed to test whether an upper limit to the energization of the belt exists.

**Fig. 5:**
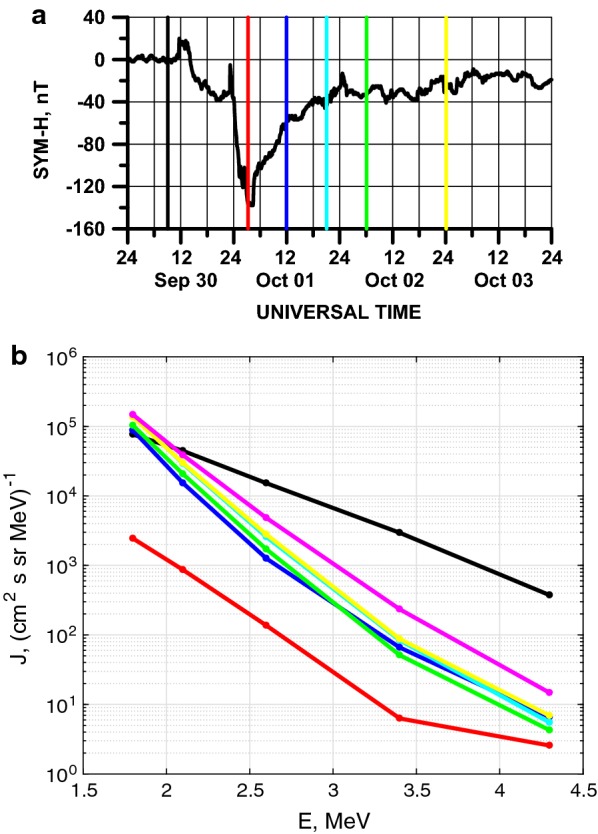
**a** SYM-H for the 1 October 2012 geomagnetic storm. Vertical color lines indicate the time of measurements of electron fluxes shown in Fig. 4b. **b** Differential electron particle fluxes measured by the ECT-REPT instrument onboard the RBSP-A (**a**). Colors correspond to the times of measurement indicated in Fig. 4a

The storms of the third type, i.e., the storms for which—according the classification of Reeves et al. (2003), Turner et al. (2013) and Moya et al. (2017)—the electron fluxes before and after the storm are nearly the same, are the most suitable for the study of the role of adiabatic effects in the electron acceleration during geomagnetic storms. In contrast to the action of wave–particle interactions mechanism of acceleration, the adiabatic variations due to temporal changes in the magnetic field easily explain the restoration of relativistic electron flux spectra with nearly the same characteristics of the spectra before the storm. To study the observed effect, we analyzed the differential electron fluxes, measured by the ECT-REPT instrument during the 30 September 2012 geomagnetic storm, shown in Fig. 3. Considering this storm event, in Fig. 5a we show the SYM-H variation for this relatively intense storm, reaching a minimum SYM-H equal to − 138 nT. Colored vertical lines correspond to the times of measurement of the electron flux as a function of energy spectra shown in Fig. 5b. For these times, the RBSP-A satellite was located at geocentric distance of 4 Re, near to 10 MLT, which is an adequate radial distance and MLT combination to obtain representative observations of the ORB characteristics. In Fig. 5b, only differential fluxes corresponding to energy channels between 1.8 and 4.3 MeV are shown. For this energy interval, the fluxes have typical characteristics of ORB relativistic electron fluxes. It is important to mention that the magenta line in Fig. 5b corresponds to the electron flux energy spectrum measured on October 6, 2012, at 17:41 UT (not shown in Fig. 5a), i.e., it corresponds to fluxes measured 3 days after the storm. As it can be easily seen in Fig. 5, the electron fluxes strongly decrease more than one order of magnitude near the storm minimum SYM-H value (red line). Then, during the recovery phase, fluxes increase returning to their initial value for ~ 2 MeV electrons. Turner et al. (2014b) shows the possibility to enhance electrons at 2 MeV and equatorial pitch angles of 90° by a factor ~ 20 by chorus waves at the analyzed geocentric distance for 10-h period (see picture 10 in their paper). This factor is increased with the energy decrease and is decreased for higher energies [which is also possible to see on the figure 10 of Turner et al. (2014b)]. Such increases of particle fluxes correspond on the order of magnitude to flux change for the period between red and blue lines on Fig. 5b when the absolute value of Dst variation is changed more than in 2 times. Turner et al. (2014b) also mentioned that “multiple energetic particle injections were observed by GOES, POES, THEMIS, and Van Allen Probes between 12:00 UT on 30 September and 12:00 UT on 1 October.” This feature means the possibility of the action of the mechanism of injection in the region of depressed magnetic field particle acceleration discussed by Tverskoy (1997). We can also see on Fig. 5b the increase in particle fluxes after 12:00 UT on 1 October when chorus activity was practically stopped in accordance with Turner et al. (2014b). So, we can attribute the recovery of the ~ 2 MeV electron fluxes to the pre-storm level observed here, and typical for storms of the third type, to the action of the adiabatic mechanism of flux variation, taking into consideration that substorm activity during recovery phase was very low (see Fig. 3). Another important argument supporting this mechanism is related to the observed changes in the hardness of the electron spectra during the recovery phase. The hardness is maximum near minimum SYM-H (red curve). Then, during the recovery phase, the spectral slopes get steeper, a behavior consistent with the quick electron acceleration during substorms observed in the main phase (see variation of the |AL| index in Fig. 3), and subsequent loss of more energetic particles during the storm recovery phase. It is important to mention that flux levels of order 10^1^ cm^−2^ s^−1^ sr^−1^ MeV^−1^ may drop below ECT-REPT noise level, especially at higher-energy channels (see, e.g., Moya et al. 2017). However, for this storm event it is clear that the spectra during the recovery phase are very close to each other, showing the stability of the electron fluxes when the magnetic field changes very slowly. Analysis of the same event by Turner et al. (2014a, b) suggests the irreversible flux “dropout” during storm main phase due to outward radial transport and magnetopause shadowing, and precipitation due to EMIC waves at *L* < 4. Suggesting the full flux “dropout” during storm main phase, it is difficult to explain the restore of particle fluxes after the storm in the ORB center at *L* ~ 4.

## Discussion

In previous works, the role of substorms in the acceleration of relativistic electrons has been analyzed mainly as a possible source of seed population of electrons, which are later accelerated by electromagnetic waves in the ULF and VLF range. However, results by Kim et al. (2015), Hajra et al. (2015), Pinto et al. (2018) and our current study show that the response of ORB electrons takes place on the timescale of a substorm; that is, it is very fast. We attribute the appearance of enhanced fluxes after geomagnetic storms to the action of adiabatic acceleration on the electron population, injected into the region in which the magnetic field has been depressed by the ring current. Thus, the recovery of the magnetic field during the storm recovery phase leads to the increase in the electron flux due to action of adiabatic acceleration. Clear verification of the role of adiabatic processes in accordance with Tverskoy (1997) suggestion requires the analysis the dynamics of ORB fluxes. Proper calculation of PSD can solve this problem. However, PSD calculation requires adequate magnetic field model (Green and Kivelson 2004). Such model has not been obtained until now (see discussion below).

Our conclusion does not coincide with well-developed model considering particle dropout due to outward radial transport and magnetopause shadowing, and precipitation due to EMIC waves and diffusion-like acceleration due to resonance with chorus waves. This model is based on the validity of Tsyganenko and Sitnov (2005) model, which is recognized by Morley et al. (2013) as the best magnetic field model. However, Van Allen Probes magnetic field observations were not done very near to the equatorial plane, where Earth’s magnetic dipole produces the main contribution to the magnetic field and all models lead to the nearly same value of the magnetic field. PSD calculations are sensitive to magnetic field values near the equatorial plane. The overstretching of non-storm time early magnetic field models was demonstrated by Reeves et al. (1997), while the overstretching of the Tsyganenko-1996 and Tsyganenko-2001 models was demonstrated by Antonova et al. (2006). In addition, Stepanova et al. (2008) using Tsyganenko and Sitnov (2005) model met with difficulties connected to singularities of this model (see figure 3 in Stepanova et al. 2008). The reason of such overstretching is the using of predefined current systems in the Tsyganenko models (Antonova et al. 2017). Later generation of models, which do not use such suggestion, stated from Tsyganenko and Sitnov (2007) has no such limitation. It is possible to hope that a new generation of magnetic field models under development—for example the models developed by Andreeva and Tsyganenko (2016) and Stephens et al. (2016)—will accurately reproduce the changes in the magnetic field during storms. However, all models are rather averaged and cannot reproduce magnetic field changes during storm time substorm injections, which require, in accordance with Morley et al. (2013), the use of event-fitted models.

The necessary condition for adiabatic mechanism to be efficient is a relatively low radial diffusion during the storm recovery phase. This diffusion is very intense during the storm main phase, when we observe very high level of magnetic and electric fluctuations, which later decrease during the storm recovery phase. Therefore, substorm injections of relativistic electrons during the storm main phase do not have a strong effect due to fast transport of particle fluxes away from the region of acceleration. On the contrary, substorm injections during the storm recovery phase are accompanied by a comparatively low radial diffusion and the electron fluxes increase when the magnetic field restores up to the pre-storm level. The existence of storms with outer radiation belt electron depletion also supports such scenario, as we found that the characteristic feature of storms resulting in depletions of the electron fluxes is the absence of substorm injections during the storm recovery phase as shown statistically in Fig. 4 and also in Fig. 2 for the 12 September 2014 magnetic storm.

The efficiency of the action of the adiabatic mechanism can be evaluated due to the analysis of the dynamics of relativistic electrons during the storms for which the electron fluxes before and after the storm are almost unchanged. It seems to us that it can be done only due to the action of the adiabatic mechanism. This behavior of electron fluxes is observed during the 30 September 2012 storm: The fluxes at *L* = 4 increase more than an order of magnitude (up to the pre-storm level) during storm recovery phase when the substorms are not observed. However, the true verification of the importance of the adiabatic acceleration mechanism requires the study of the evolution of the electron phase space density during each event. Another important feature is the existence of local holes in the magnetic field that appear due to local increases of plasma pressure at the equatorial plane even during relatively weak levels of geomagnetic activity (Vovchenko and Antonova 2017). Formation of a magnetic field depression near to the maximum of the ring current in such a case is a natural suggestion. However, the value of this kind of depression is not well known. The trapping of particles in such magnetic holes may be an important feature of storm dynamics, not properly studied yet.

Another unsolved problem is the appearance of relativistic injections during substorms. Acceleration of relativistic electrons during probable mechanism of such very quick acceleration is the interaction of electrons with high-frequency electrostatic waves. However, this subject is beyond the scope of this work. For our study, it is only important that such acceleration is really observed.

## Conclusions

In this study, we have analyzed Van Allen Probes observations of the outer radiation belts electrons during 78 magnetic storms with different intensity. Storms resulting in the enhancement, depletion or no change in relativistic electron fluxes were identified. We show that substorm activity during storm recovery phase—quantified by the AL index—is observed for all storms with flux enhancement. Such activity was not practically observed for storms with flux depletion and no-change storms. This finding shows that substorm injections in the region of the magnetic field, depressed by the ring current, are a very important feature of storm time dynamics.

We also analyzed changes in the differential fluxes of relativistic electrons during the 30 September 2012 geomagnetic storm, selected as an example of a no-change storm. Substorm activity at this storm was observed only during the main phase and at the very beginning of the recovery phase. This substorm activity leads to the formation of comparatively low relativistic electron fluxes with low slope spectra. During storm recovery phase, the increase in relativistic flux intensity and increase in slope is observed. Such feature is difficult to explain, suggesting the slow acceleration of relativistic electrons by whistler-mode chorus waves during storm recovery phase. Such acceleration cannot also explain the restoration of relativistic electron fluxes with energy ~ 2 MeV to the pre-storm level. We suggest that the only reasonable explanation of such feature is the adiabatic acceleration of electrons due to the restore of the magnetic field. The support of this suggestion requires measurements of the magnetic field change at the equatorial plane, which is desirable for future experiments.

In general, this work clarifies the role of auroral processes, especially substorms, in the acceleration of the ORB electrons. Additionally, to the results reported by Antonova and Stepanova (2015), we found new evidences supporting the Tverskoy (1997) and Antonova (2006) theory of relativistic electron acceleration. However, the verification of all predictions of this theory will require extensive study.

## Abbreviation

ORB: outer electron radiation belt
